# Direct Analysis of Incorporation of an Anticancer Drug into DNA at Single-Molecule Resolution

**DOI:** 10.1038/s41598-019-40504-x

**Published:** 2019-03-07

**Authors:** Takahito Ohshiro, Yuuki Komoto, Masamitsu Konno, Jun Koseki, Ayumu Asai, Hideshi Ishii, Masateru Taniguchi

**Affiliations:** 1 The Institute of Scientific and Industrial Research, Osaka University, 8-1 Mihogaoka, Ibaraki Osaka, 567-0047 Japan; 2 Department of Frontier Science for Cancer and Chemotherapy, Graduate School of Medicine, Osaka University, 2-2 Yamada, Suita, Osaka, 565-0871 Japan; 3 Department of Medical Data Science, Graduate School of Medicine, Osaka University, 2-2 Yamanda, Suita, Osaka, 565-0871 Japan

## Abstract

Identifying positions at which anticancer drug molecules incorporate into DNA is essential to define mechanisms underlying their activity, but current methodologies cannot yet achieve this. The thymidine fluorine substitution product trifluridine (FTD) is a DNA-damaging anticancer agent thought to incorporate into thymine positions in DNA. This mechanism, however, has not been directly confirmed. Here, we report a means to detect FTD in a single-stranded oligonucleotide using a method to distinguish single molecules by differences in electrical conductance. Entire sequences of 21-base single-stranded DNAs with and without incorporated drug were determined based on single-molecule conductances of the drug and four deoxynucleosides, the first direct observation of its kind. This methodology may foster rapid development of more effective anticancer drugs.

## Introduction

Millions of people in the world suffer will suffer from cancer in their lifetime, making development of novel anticancer drugs a global concern^1^. Moreover, ongoing development of new anticancer drugs is essential to respond to drug resistance issues. Patients with refractory colorectal tumors have been successfully treated with TAS-102^2^, a formulation of trifluridine (FTD) and tipiracil hydrochloride (TPI) at a molar ratio 1:0.5, after at least one standard chemotherapy^3–6^.

Mechanistically, FTD is sequentially phosphorylated by thymidine kinase to form FTD triphosphate (FTD-TP), which, during DNA replication, is incorporated into DNA by DNA synthetase in place of thymidine monophosphate (dTMP)^5,6^. As part of TAS-102, TPI inhibits FTD-degrading enzymes, including thymidine phosphorylase, and as a result maintains high blood levels of FTD. FTD cannot base pair with adenine via hydrogen bonding, and thus its incorporation perturbs transcription factor binding, promoting aberrant gene expression and ultimately resulting in cell death.

DNA melting point analysis shows that inclusion of FTD in DNA duplexes destabilizes helical structure, and that methodology has been used to evaluate abnormal hydrogen bonding in FTD-incorporated DNA^7^. However, since currently used DNA sequencing technology cannot directly detect FTD in DNA, it remains unclear where and how FTD is incorporated into a strand. This knowledge is essential to develop novel DNA-damaging drugs as cancer therapies.

**Figure 1:**
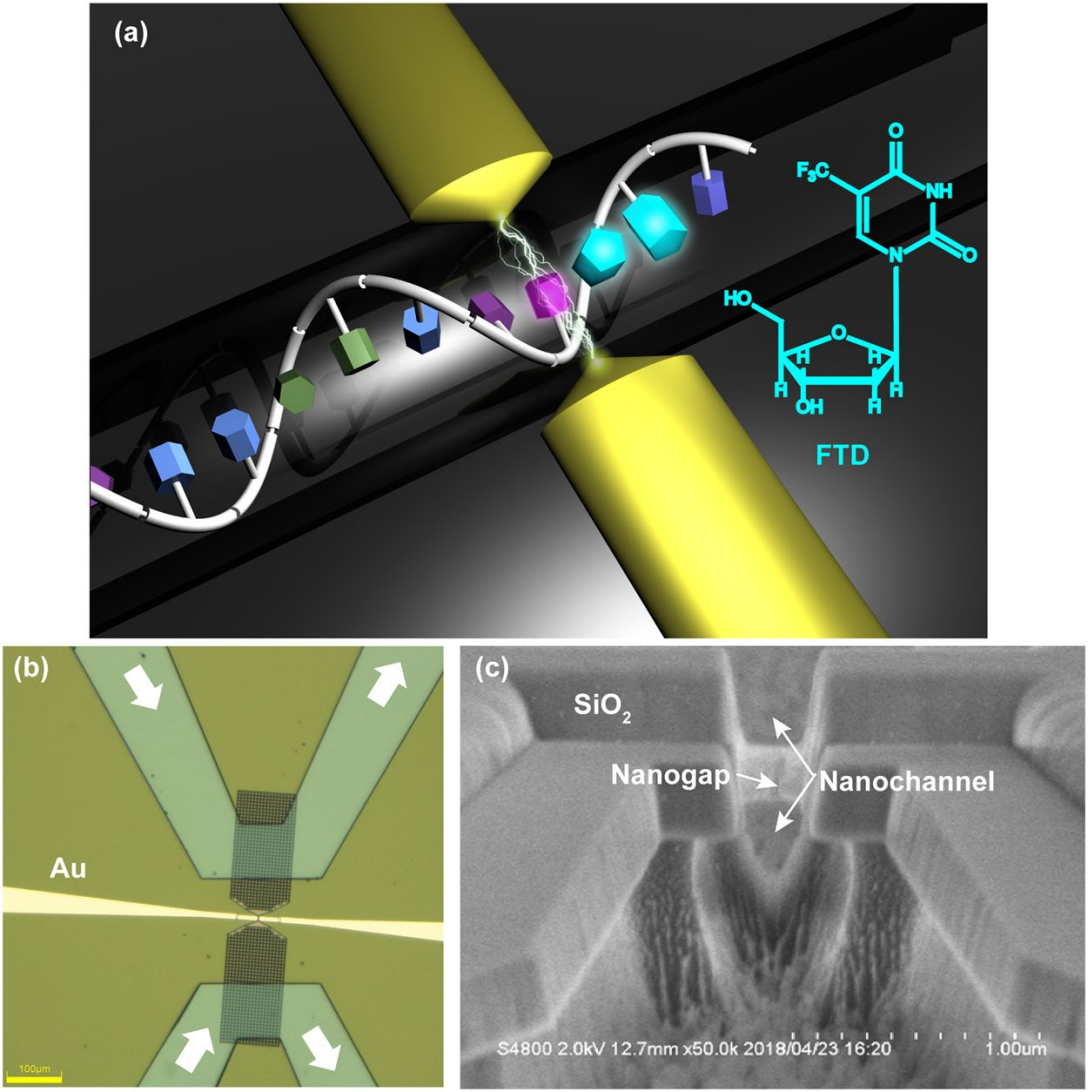
Single-molecule quantum sequencing method using nanogap electrodes. (**a**) Operating principles of single-molecule quantum sequencing. Single molecules were identified by tunneling currents flowing between electrodes through either single nucleotides or drug molecules. At right is the structure of the anticancer drug FTD. (**b**) Optical microscope image of a measurement chip with integrated microchannels, nanochannels, and nanogap electrode. Yellow indicates gold (Au) electrodes. White arrows indicate direction of the aqueous solution flowing through microchannels. Microchannels are connected to the SiO_2_ nanochannel (gray section), and the nanogap electrode is located in the narrow section of the nanochannel. Yellow scale bar: 100 nm. (**c**) Scanning electron microscope image of nanochannels and nanogap electrodes. Image is an enlargement of the stenotic section of the nanochannel seen in. (**b**) Nanogap electrodes are coated with SiO_2_, except for the tip, and have a hollow structure.

Here, we directly determine the position of FTD in single-stranded DNA using a single-molecule quantum sequencing method (Fig. 1(a))^8^. This method is useful to determine DNA base sequences and peptide amino acid sequences based on single-molecule conductance obtained from tunneling current flowing between electrodes through single molecules. In comparison with nanopore sequencers, which determine base sequences using changes in the ionic current, this method is advantageous because it can directly determine the base sequences in RNAs and the amino acid sequences in peptides^8^. This method can also directly distinguish between chemically-modified bases and amino acids, which can serve as important disease markers^8^.

We performed single-molecule quantum sequencing using nanoelectrodes with a 0.7 nm inter-electrode gap fabricated by feedback control based on current flow between electrodes (Sample preparation and Signal detection in Methods). To reduce interference caused by ionic currents in an aqueous solution, we coated gold electrodes with an insulating SiO_2_ film and applied a voltage of 0.1 V between nanoelectrodes. Furthermore, to effectively transport DNA between nanoelectrodes, SiO_2_ was processed into a nanochannel, which was connected to microchannels formed from polydimethylsiloxane (PDMS; Figs 1(b,c) and S1). Nanochannel height and width were 300 and 200 nm, respectively, and the inside of the nanochannel was made hydrophilic by oxygen plasma treatment.

Using this setup, we detected and defined FTD position in DNA using the following procedure. First, we assayed an aqueous solution containing FTD alone to determine FTD single-molecule conductance. We then assessed single-molecule conductance–time profiles in aqueous solutions containing the single-stranded oligonucleotide 5′-TCACAAGTTAGAGACAAGCCT-3′ or a comparable oligo containing FTD (F) at the 8th and 9th thymines (5′-TCACAAGFFAGAGACAAGCCT-3′), and determined partial base sequences by constructing single-molecule conductance histograms of the four deoxynucleosides and FTD. Subsequently, we determined entire DNA sequences with and without FTD using an assembly method based on the presence of overlapping sections of partial sequence at the margins^9,10^. DNA sequences revealed in this analysis exhibited homology to regions of the tumor suppressor gene p53 gene^11^.

**Figure 2:**
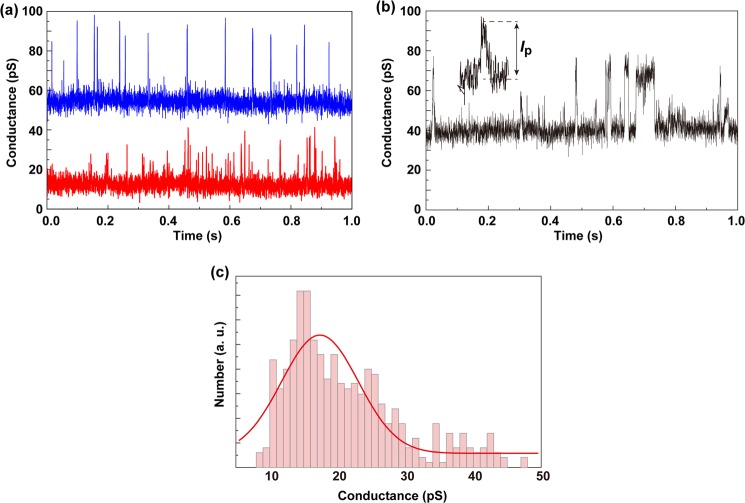
Single-molecule discrimination between thymidine and FTD. (**a**) Typical single-molecule conductance–time profiles obtained in aqueous solutions containing thymidine (blue) or FTD (red). (**b**) Typical single-molecule conductance–time profile obtained in an aqueous solution of thymidine and FTD mixed at a 1:5 ratio. Single-molecule conductance–time waveforms are characterized by the maximum current (*I*_p_). Lower and higher conductances correspond to FTD and thymidine, respectively. (**c**) Histogram of *I*_p_ obtained by FTD measurements. Data is fitted to a Gaussian function (red line).

Next, to determine single-molecule conductances of FTD and four deoxynucleosides, we evaluated current–time profiles of each in aqueous solution and obtained waveforms characterized by maximum current (*I*_p_) and current duration (Fig. 2(a)). Analysis of histograms constructed from 450 *I*_p_s showed the FTD peak single-molecule conductance to be 17.9 pS, while corresponding values of deoxyguanosine (G), deoxyadenosine (A), deoxycytidine (C), and thymidine (T) were larger: 86.7, 66.8, 59.5, and 39.1 pS, respectively^9,10^. Electronic states of the four deoxyribonucleotides and FTD monophosphate (FTD-MP) were calculated using density functional theory; energies of the highest occupied molecular orbitals (HOMOs) were −6.19 eV (G), −6.45 eV (A), −6.52 eV (C), −6.87 eV (T), and −7.99 eV (FTD), consistent with the single-molecule conductance values. We conclude that the strong electron-withdrawing properties of the FTD fluorine atom decrease HOMO energy level. Since energy gaps between the lowest unoccupied molecular orbitals (LUMOs) and the Fermi level are larger than those between HOMOs and the Fermi level (when Fermi energy of the electrode is assumed to be 5 eV), we conclude that electrons are transported through electronic states derived from HOMOs of each molecule^12–15^.

Given that single-molecule conductance depends on the distance between electrodes and their surrounding environment, we used relative single-molecule conductances normalized by the largest single-molecule conductance, namely deoxyguanosine, rather than absolute values to distinguish between the four deoxyribonucleotides and FTD-MP. When we measured current–time profiles of a solution in which the FTD:thymidine ratio was 1:5, we obtained spike-like signals similar to those of individual deoxynucleosides (Fig. 2(b)). We then undertook histogram analysis and found that relative single-molecule conductances yielded higher and lower conductance signals corresponding to thymidine and FTD, respectively. In this analysis, bases were stochastically assigned to single current–time waveforms based on the observation that relative single-molecule conductance histograms corresponded to probability density functions of conductances of each nucleotide. These results indicate that FTD can be identified in a DNA strand as a fifth deoxynucleoside in addition to A, C, G, and T.

**Figure 3:**
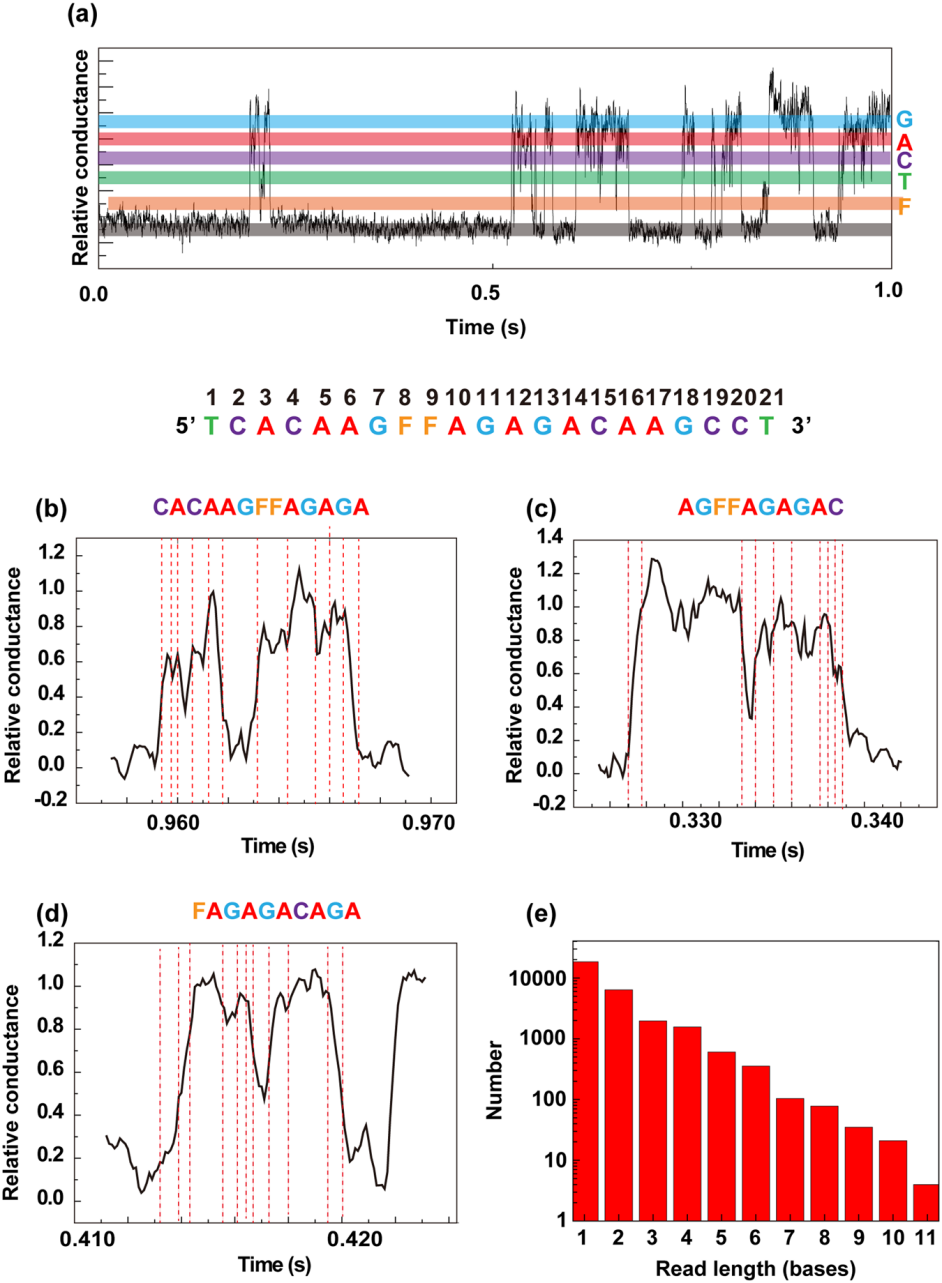
Partial sequencing of FTD-incorporated DNA. (**a**) Typical relative single-molecule conductance–time profile obtained in an aqueous solution of single-stranded, FTD-incorporated DNA. Color bands provide visual guide to G, A, C, T or FTD (F) profiles. (**b**–**d**) Relative single-molecule conductance–time waveforms of three partial sequences. Red dashed lines indicate transition points between adjacent bases. (**e**) Histogram showing length of partial sequences obtained by assaying single-stranded DNA containing FTD.

To determine partial sequences in DNA strands, we assessed current–time profiles of aqueous solutions of single-stranded 5′-TCACAAGTTAGAGACAAGCCT-3 DNA and corresponding single strands of FTD-incorporated 5′-TCACAAGFFAGAGACAAGCCT-3 (Fig. 3(a)). We observed similar current–time waveforms among the four deoxynucleosides and FTD. We determined partial sequences of DNA strands in an automated assay using a home-made base sequence program^9,10^ employing relative single-molecule conductance histograms of each deoxynucleoside and FTD. By dividing the current–time waveform every 0.5 ms and integrating the probability density function of the four deoxynucleosides and FTD in 0.5 ms sections, we were able to assign the molecular species with the maximum probability within the interval to the waveform. The empirically determined minimum time that a single deoxyribonucleotide remains in the vicinity of the nanogap electrodes is reportedly 0.5 ms^9,10^. The total number of partial base sequences was 29,628, a number that decreased exponentially with read length and had a maximum value of 11 (Fig. 3(e)).

DNA behavior is strongly influenced by Brownian motion, as DNA moves back and forth between electrodes and passes between electrodes in a single direction. Consequently, the current duration corresponding to a single base increases with reciprocating motion^9,10^. Figure 3(b,c) show typical current–time profiles of the partial sequences CACAAGFFAGAGA and AGFFAGAGAC, respectively, in which sequences were read out in a single direction. The current–time waveform of Fig. 3(d) was determined to be FAGAGACAGA. Reciprocating motion would allow for two possibilities: one, that the last A was misread, and the other, that A was read out when DNA was read in the reverse direction at G18, after which DNA separated from the electrode.

**Figure 4:**
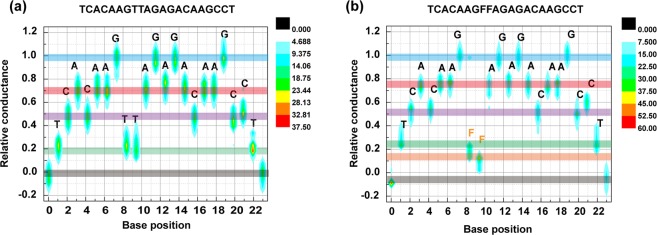
Whole base sequence determination of single-stranded DNA containing FTD moieties. (**a**,**b**) Heat maps of relative single-molecule conductances of whole base sequences of oligonucleotides without (**a**) and with (**b**) FTD molecules. Light blue, pink, purple, green, and orange bands serve as visual guides showing single-molecule conductances of deoxyguanosine, deoxyadenosine, deoxycytidine, thymidine, and FTD, respectively.

Finally, in order to demonstrate direct analysis of incorporation of an anticancer drug into DNA at single-molecule resolution, sequences of DNA with and without FTD were automatically determined using an assembly method in which shared regions of partial sequences of nine or more bases comprised a border, and overlapping partial sequences then used to construct an entire sequence^9,10^. As clearly indicated in single-molecule conductance heat maps, the relative single-molecule conductance of FTD was less than that of thymidine (Fig. 4(a,b)). This result is consistent with single-molecule conductances of the four deoxynucleosides and FTD and confirms that FTD is incorporated into DNA. For bases other than FTD, we obtained relative single-molecule conductances at the same positions in both 21-base oligonucleotides whose sequences differed only at bases 8 and 9.

It is challenging to obtain high determination accuracy of continuous sequences in a DNA sequencer. Here, there are continuous sequences of TT and FF in the 8th and 9th positions. Sequencing of consecutive duplicate bases was executed according to the following procedure: First, the relative single-molecule conductances of T and F were determined based on the mode of the monomer histograms. Next, T or F was assigned based on the obtained conductance values at the 8th and 9th positions. At this time, the relative single-molecule conductance determination of T or F was assigned based on the mode of the monomer histograms. Since the conductances of the 8th and 9th positions were practically indistinguishable, the base species at positions 8 and 9 were assigned according to the following method: Only partial sequences with 6 bases or more, including the 8th or 9th position, were used as correct sequences for assembly. If the correct assembly sequence contained an 8th or 9th position, the obtained conductance was assigned to 8th and 9th positions. When the 8th position or the 9th position was the terminal base and the reading direction was from the 3′ end to the 5′ end, the obtained conductance value was assigned to the 9th position. On the other hand, when the 8th or 9th position was the terminal base and the reading direction was from the 5′ end to the 3′ end, the obtained conductance value was assigned to the 8th position.

The precision of this method could be obtained using standard DNA sequencing accuracy evaluation methods: using *p* values and signal numbers (*N*) (degree of sequence reading overlapping, see Fig. S3)^9,10^. Analysis of DNA containing FTD with continuous sequences at positions 8 and 9 by this method yielded 364 and 562 signal numbers at positions 8 and 9, respectively. The average base reading accuracy obtained from the one current-time profile was 79.1% and 80.8% at the 8th and 9th positions, respectively The number of signals required to achieve an accuracy of >99.99999% were 13 and 12, respectively; therefore, an accuracy of >99.99999% was achieved. On the other hand, for DNA without FTD, the number of signals at positions 8 and 9 were 8948 and 13439, respectively. The average reading accuracy of bases obtained from the one current-time profile was 83.3% and 77.0% at the 8th and 9th positions, respectively. The number of signals required to achieve an accuracy of >99.99999% were 11 and 14 for the 8th and 9th positions, respectively; therefore, an accuracy of >99.99999% was achieved.

Using methodology based on single-molecule conductances, we determined the entire sequence of single-stranded DNA consisting of 21 bases and of a corresponding oligo in which two thymidines were replaced with the anticancer drug FTD. This is the first time that a drug incorporated into single-stranded DNA has been detected and its substitution positions defined. We anticipate that other anticancer drug molecules can be detected using this method, which reads differences in electronic states of single molecules by tunneling currents. This single-molecule quantum sequencing method should be a powerful tool to investigate how and where anticancer drugs incorporate into DNA strands.

In this paper, the single-molecule quantum sequencer could read the full length of the 21-base strand tested; however, the frequency with which 21 bases could be read was extremely low. Therefore, to read longer base sequences, it will be necessary to develop flow-dynamics control techniques to flow DNA and RNA with high efficiency into the nanogap^16,17^ Additionally, for sending long single-stranded DNA or RNA through the nanogap, it will be necessary to prepare nanogap electrodes in nanopores or nanochannels that can control the space through which the DNA and RNA can pass. The development of nanoelectrodes in nanochannels and flow-dynamics control technology for DNA and RNA by electrophoresis is currently underway in our lab. At the same time, we are developing nanostructures with nanogap electrodes in nanopores.

## Methods

### Sample preparation

We purchased trifluridine (FTD) and thymidine from Sigma-Aldrich and obtained oligonucleotides (5′-TCACAAGFFAGAGACAAG CCT-3′, 5′-TCACAAGTTAGAGACAAGCCT-3′) from Takara Bio, Inc., and Hokkaido System Science Co., Ltd. The oligonucleotide samples were purified by high performance liquid chromatography (HPLC). Nucleotide samples were diluted in MilliQ water, and the total nucleotide concentration adjusted to 1 μM for electrical measurement. Prepared sample solutions were inserted into the solution chamber of a nanofabricated mechanically controllable break junction (MCBJ) device^18,19^, which was produced by a nanofabrication technique. The detailed fabrication procedure is described in supporting information.

### Signal detection

A nanofabricated MCBJ was used for electrical measurement. The MCBJ substrate was bent, and the junction was mechanically broken to form a pair of gold nanoelectrodes. After reconnecting the gold junction, a constant DC bias voltage of 0.1 V was applied, and the substrate was gradually bent using a piezo-actuator. While breaking the junction, the junction conductance (*G*) was monitored using a picoammeter (Keithley 6487). A series of conductance jumps of the order of *G*_0_ = 2*e*^2^/*h* (where *e* and *h* are the electron mass and the Planck constant, respectively) was observed, and the final conductance was 1 *G*_0_. Several seconds after the 1 *G*_0_ state was obtained, the single gold atom contact was naturally ruptured, resulting in a pair of electrodes. The gap width was controlled by tuning the piezo-voltage, and the electrode gap was set to an optimal value for each nucleotide measurement. The current–time profile was acquired at a sampling rate of 10 KHz using a transimpedance amplifier with a band of 100 KHz and a PXI-4081 digital multimeter (National Instruments) at a DC bias voltage of 0.1 V. On the basis of the observed current profiles, gap width was maintained at the optimal value during a whole run as follows: every 0.5 s, the baseline current was defined as the mode value of the observed current–time profile of 5,000 data points. The baseline current in the observed current profile represents the tunnel current of the gap at the time. The tunnel current (*I*) can be expressed by the equation

$${\rm{I}}\propto \exp (-const.\times d\sqrt{\phi }),$$

where *d* is the gap width and *φ* is the work function of the gap electrode.

From this equation, the tunnel current is exponentially proportional to gap width. To keep the set gap width, the baseline current was maintained by adjusting the piezo-voltage during the whole run.

### Signal pickup

Before signal analysis, noise signals were picked up from nucleotide signals in the current–time profiles. Criteria for signal pickup are as follows. First, the peak value *I*_*p*_ in the signal is defined as the local maximum ( = maximum current − baseline current), where the baseline current (*I*_*baseline*_) at the peak time in the current–time profiles was defined as the simple moving average of the nearest 2,000 data points. Secondly, the first and second signal thresholds are defined as *T*_1_ ( = baseline + 6 × region standard deviation (*STD*_*region*_)) and *T*_2_ ( = baseline + region standard deviations (*STD*_*region*_)), where the region standard deviations (*STD*_*region*_) are defined as the mode value of the standard deviation of 20 data currents ( = current − baseline current) in the nearest 2,000 data points. Finally, in current–time profiles, the signal data point region is determined by the signal start time (*t*_start_) and the signal end time (*t*_end_), where *t*_start_ is defined as the data point where the current exceeds *T*_1_ and *t*_end_ is defined as the data point where the current falls below *T*_2_. For signal detection of each sample nucleotide, at least 10 MCBJ samples were used, and >1,000 signals were obtained for each sample.

### Signal assignment

Typical conductance–time profiles are shown in Fig. 3(a). Current histograms were constructed from *I*–*t* profiles using data obtained. The lowest conductance peak current is assigned to the baseline current, and other conductance peaks in histograms corresponded to base species in the signals, as shown in previous reports^9,10^. The assignment procedures were as follows. First, the relative conductances for each mononucleotide (G, A, C, T, or FTD) were determined. In this study, the FTD peak value was found to be 17.9 pS from the Gaussian fitting peak of the histogram (Fig. 2(c)) so that, combined with analysis from previous reports^9,10^, we determined conductance of a mononucleotide relative to guanine conductance to be 0.77 for A, 0.69 for C, 0.45 for T, and 0.21 for FTD. Secondly, each base probability was calculated for the time in current–time profiles from the equation:

$$P(x)=\frac{1}{\sigma \sqrt{2\pi }}exp(-\frac{{(x-\mu )}^{2}}{2{\sigma }^{2}}),$$

where *x* is the conductance relative to the guanine conductance (maximum conductance peaks), *μ* is the relative conductance peak value for a specific base, and *σ* is the standard deviation for the signal region data point.

Finally, on the basis of the calculated maximum values among probabilities for each base species and baseline, the base molecule type or baseline was determined for each time in the time profile.

### Quantum chemical calculations

All calculations were performed using the program DMOL^3^ based on density functional theory^20,21^. We employed the GGA/BLYP method^22,23^ with a standard double numerical basis set for all electrons. Structural optimization was conducted under convergence conditions where the self-consistent field, energy, maximum force, and maximum displacement were 1 × 10^−4^ Hartree, 1 × 10^−4^ Hartree, 0.02 Hartree/Å, and 0.05 Å, respectively. All phosphate groups were hydrogen-terminated. The total charge was zero, with a singlet spin multiplicity. The charges of each nucleotide were calculated from the Mulliken charge.

## Supplementary information

SupplementaryInformation
