# Supplementary material for: Direct Analysis of Incorporation of an Anticancer Drug into DNA at Single-Molecule Resolution
Source: Sci Rep. 2019 Mar 7;9:3886. doi: 10.1038/s41598-019-40504-x (PMC6405915; doi:10.1038/s41598-019-40504-x)
Supplement: Supplementary file 1 — SupplementaryInformation [file 41598_2019_40504_MOESM1_ESM.docx]

**Supplementary Information for**

**Direct Analysis of Incorporation of an Anticancer Drug into DNA at Single-Molecule Resolution**

Takahito Ohshiro, Yuuki Komoto, Masamitsu Konno, Jun Koseki, Ayumu Asai

Hideshi Ishii, and Masateru Taniguchi

Supplementary Information includes:

1. Supplementary Figures (S1-S3)

**
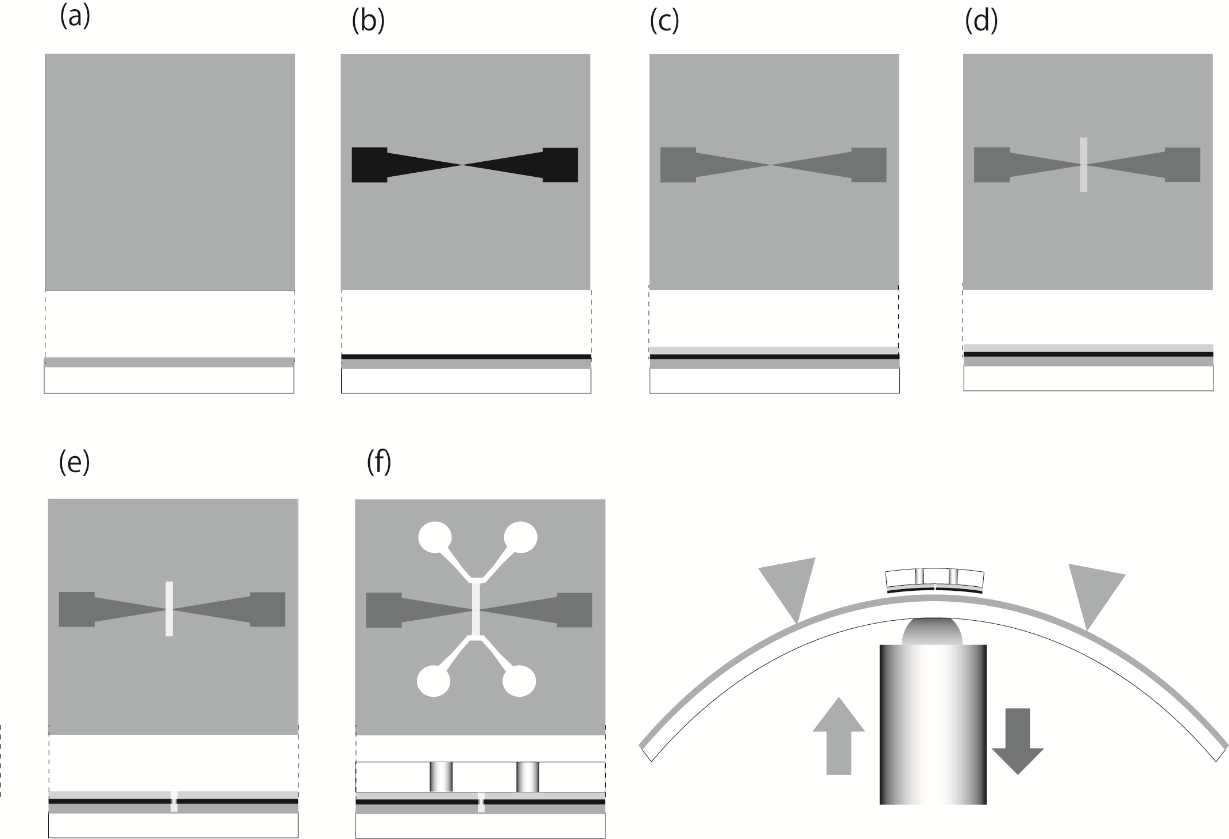
**

**Figure S1. MCBJ chip fabrication.** A nanofabricated, mechanically-controllable break junction (MCBJ) was employed to form nucleotide-sized electrode gaps. MCBJ fabrication was as follows. (a) We first coated a silicon substrate with a thin polyimide layer for electrical insulation. (b) We then fabricated a gold nanojunction by standard electron-beam lithography and using a subsequent lift-off process. (c) A thin silicon oxide layer was then coated onto the gold junction for electrical insulation by chemical vapor deposition. Electrical insulation suppresses electrical noise during current measurements. (d) We then overlaid a fluid pattern onto the gold nanojunction by electron-beam lithography. (e) The junction and fluid pattern were then exposed to isotropic reactive ion etching using CF_4_/O_2_ gas to remove underlying silicon oxide and polyimide layers, resulting in a patterned fluid and free-standing gold nanobridge. (f) The fabricated fluid-integrated gold junction device was then fused to a PDMS cover, which contained microfluid and a chamber for the sample solution. The MCBJ sample was mounted in a three-point bending configuration. The substrate was bent and the junction mechanically broken to form a pair of gold nanoelectrodes. We then monitored junction conductance using a Keithley 6487 picoammeter (Keysight) at a DC bias voltage of 0.1 V.


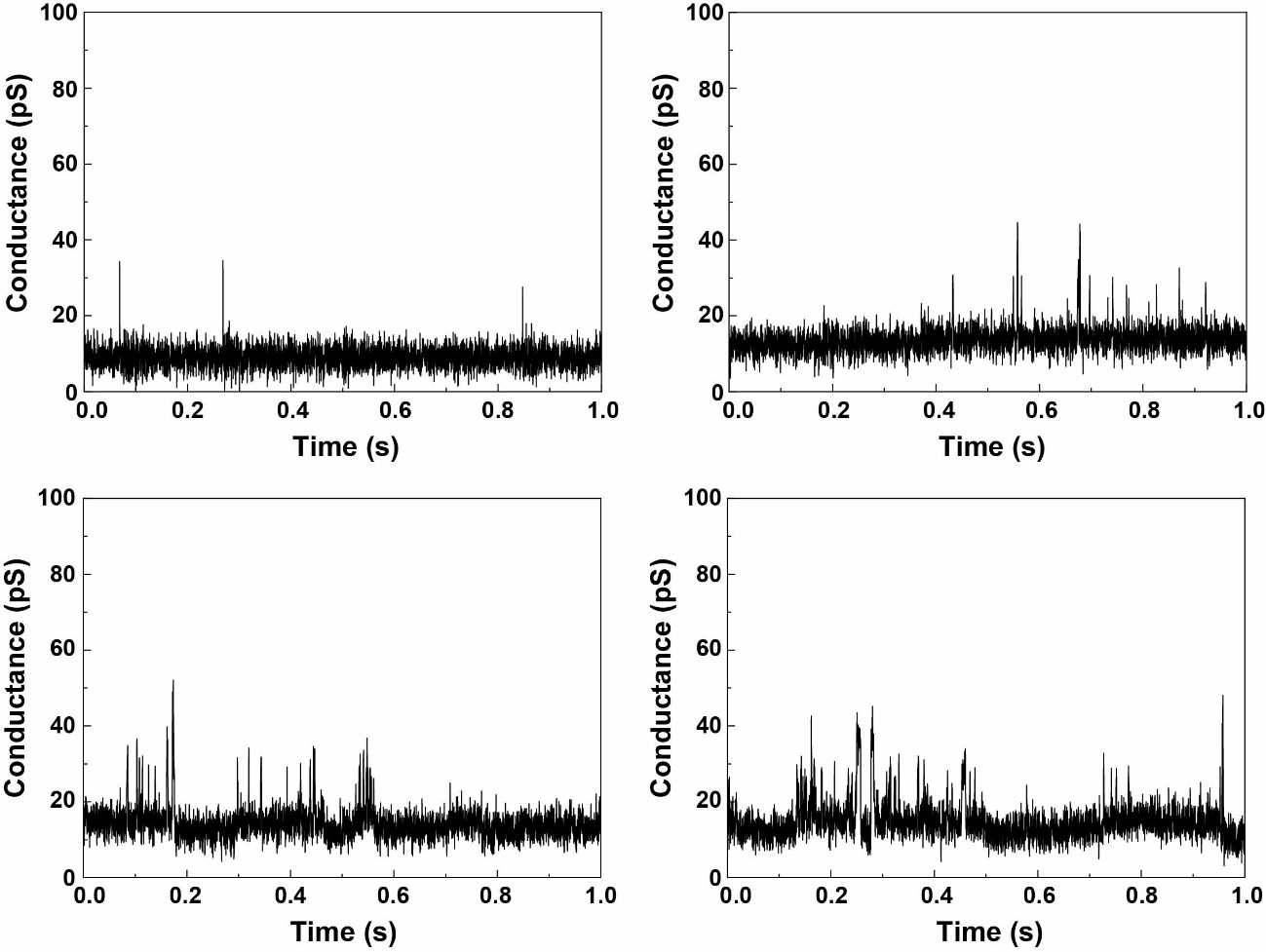


**Figure S2. Typical single-molecule conductance profiles obtained in aqueous solution containing FTD.**


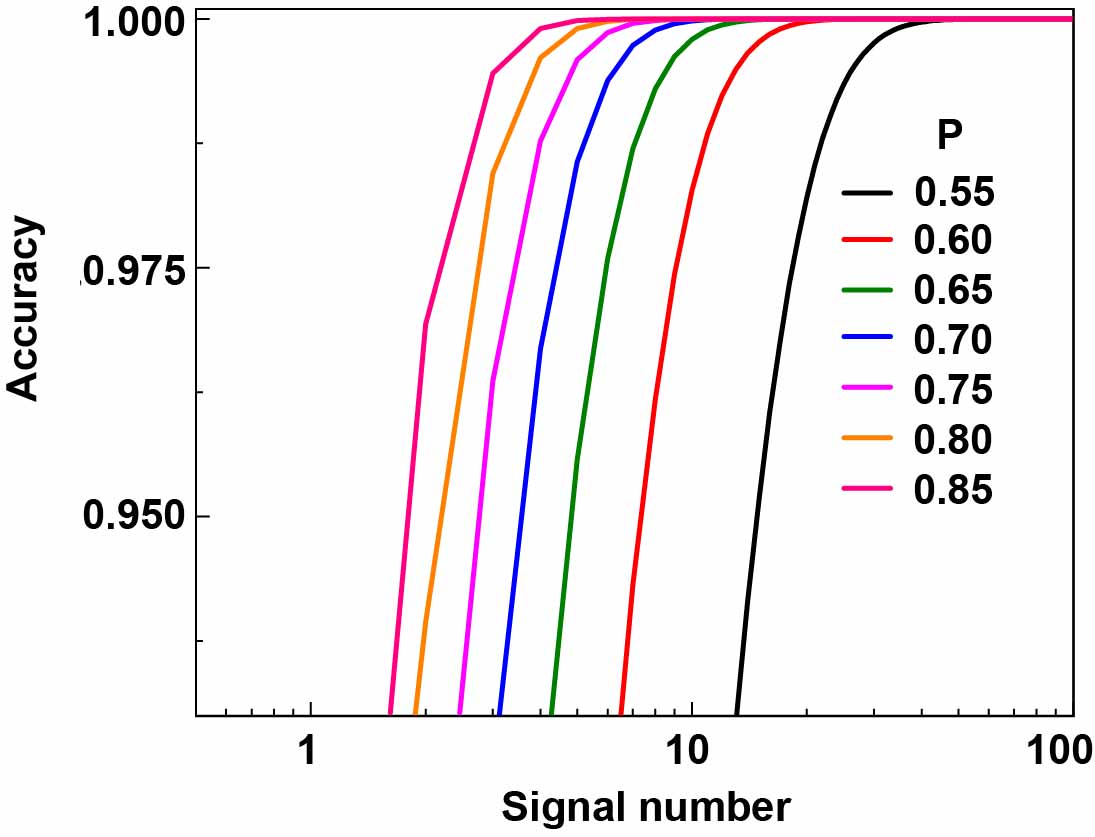


**Figure S3. Signal number dependence of accuracy.** When the assignment accuracy and the number of signals of one base in the base sequence are *p*(x) and *N* respectively, the read accuracy (*P)* of the base sequence can be given by the following equation:

$$P=exp\left\{ -\left( \frac{\bar{p}(x)}{1-\bar{p}(x)} \right)^{-N} \right\}$$

In this experiment, the average precision $\bar{p}(x)$ of F in the x = 8th and 9th positions were 0.791 and 0.808, respectively; therefore the number of signals required to achieve *P*　> 99.99999% were 13 and 12, respectively. In this experiment, the number of signals used for analysing the 8th and 9th positions were 364 and 562, respectively; therefore, an accuracy of > 99.99999% was achieved. The average precision of T in the 8th and 9th positions was 0.833 and 0.770, respectively; therefore, the number of signals required to achieve *P*　> 99.99999% were 11 and 14, respectively. In this experiment, the number of signals used for analysing the 8th and 9th positions were 8948 and 13439, respectively; therefore, an accuracy of > 99.99999% was achieved.
